# Transcranial Magnetic Stimulation Across the Lifespan: Impact of Developmental and Degenerative Processes

**DOI:** 10.1016/j.biopsych.2023.07.012

**Published:** 2023-07-29

**Authors:** Lindsay M. Oberman, Alberto Benussi

**Affiliations:** National Institute of Mental Health Intramural Research Program, National Institutes of Health, Bethesda, Maryland; Neurology Unit, Department of Clinical and Experimental Sciences, University of Brescia, Brescia, Italy.

## Abstract

Transcranial magnetic stimulation (TMS) has emerged as a pivotal noninvasive technique for investigating cortical excitability and plasticity across the lifespan, offering valuable insights into neurodevelopmental and neurodegenerative processes. In this review, we explore the impact of TMS applications on our understanding of normal development, healthy aging, neurodevelopmental disorders, and adult-onset neurodegenerative diseases. By presenting key developmental milestones and age-related changes in TMS measures, we provide a foundation for understanding the maturation of neurotransmitter systems and the trajectory of cognitive functions throughout the lifespan. Building on this foundation, the paper delves into the pathophysiology of neurodevelopmental disorders, including autism spectrum disorder, attention-deficit/hyperactivity disorder, Tourette syndrome, and adolescent depression. Highlighting recent findings on altered neurotransmitter circuits and dysfunctional cortical plasticity, we underscore the potential of TMS as a valuable tool for unraveling underlying mechanisms and informing future therapeutic interventions. We also review the emerging role of TMS in investigating and treating the most common adult-onset neurodegenerative disorders and late-onset depression. By outlining the therapeutic applications of noninvasive brain stimulation techniques in these disorders, we discuss the growing body of evidence supporting their use as therapeutic tools for symptom management and potentially slowing disease progression. The insights gained from TMS studies have advanced our understanding of the underlying mechanisms in both healthy and disease states, ultimately informing the development of more targeted diagnostic and therapeutic strategies for a wide range of neuropsychiatric conditions.

Transcranial magnetic stimulation (TMS) is increasingly being used in clinics and laboratories to study and treat a range of child, adult, and geriatric neurological and psychiatric disorders. In this context, it is crucial to carefully consider how development, aging, and pathophysiology may affect the safety of or response to TMS in these populations. The existing literature generally indicates that TMS is well tolerated across different age groups, with most reported side effects being mild to moderate, such as headaches and scalp discomfort (1–3). More serious adverse events, including seizure and syncope, have been rare (<1% of participants) even in disorders with higher seizure risk (e.g., epilepsy, stroke, and neurodevelopmental disorders). More information regarding the safety profile of TMS across the age span can be found in the Supplement.

Different stimulation parameters and types of protocols are used to probe or lead to short- or long-term modulation of specific neurophysiological mechanisms [for a review, see (4,5)]. Single-pulse TMS is used to measure cortical excitability, central motor conduction time, contralateral cortical silent period (cSP) (a measure of GABA_B_ [gamma-aminobutyric acid B] receptor activation), and corticospinal and corticocortical associative plasticity (when paired with a peripheral or central stimulation with specifically timed interstimulus intervals) and to map effective connectivity between the stimulated region and other brain areas (6). TMS can also be applied in pairs of pulses (i.e., paired-pulse stimulation) to study intracortical inhibition and facilitation (7,8). The effects of single-pulse or paired-pulse TMS paradigms last less than a second and are thus often recorded using continuous electromyographic (for primary motor cortex) or electroencephalographic (for other cortical location) recordings. TMS can also be applied in trains of regularly repeating TMS pulses (i.e., repetitive TMS [rTMS]). rTMS can be applied at various stimulation frequencies (e.g., 1–10 Hz) and patterns (e.g., theta burst stimulation [TBS]) (9). These rTMS protocols can be used to probe and induce various forms of plasticity and induce a therapeutic neuromodulatory effect. More details on these protocols can be found in Table 1 and in the Supplement.

However, the physiological and clinical effects of TMS are not simply a matter of the stimulation parameters but also of how the stimulation is received and processed in the brain. The degree and direction of neurophysiological effects of TMS are influenced by the state of excitability of the targeted cortical region and the degree of structural and functional connectivity across the targeted network. There are considerable changes in these neurophysiological states across the lifespan during both child development and older age such as maturation of GABAergic and glutamatergic neurons during childhood and adolescence (10); dopaminergic (11) and hormonal changes that occur during adolescence; and decreased dopaminergic, serotoninergic, and cholinergic transmission later in life (12,13). Development- and experience-dependent changes in functional and structural network architecture also occur across the age span (14–16). Intrinsic resting brain oscillations vary with age, potentially influencing cortical receptivity to neurostimulation (17). Illness and/or injury can affect both the neurophysiological state of the targeted region and the functional and structural connectivity properties of networks (18–20).

Here, we summarize the literature on the use of TMS to study and treat pediatric, neurodevelopmental, and degenerative psychiatric and neurological disorders. Data are presented from studies in which TMS was used to probe brain mechanisms underlying these disorders as well as development of therapeutic rTMS protocols designed to modulate these putative mechanisms to improve clinical symptom domains.

## NORMAL DEVELOPMENT AND HEALTHY AGING

TMS has been used to investigate neurophysiological mechanisms of excitability and plasticity throughout development and healthy aging. Single pulses of TMS over the primary motor cortex in children show higher resting motor thresholds (RMTs) than in adults, likely due to development of corticospinal tract myelination (21,22). Despite requiring higher stimulation intensities, children’s motor evoked potentials (MEPs) are typically smaller, delayed in latency, and polyphasic compared with adults (23,24). Combining TMS with electroencephalography (EEG) to evaluate cortical excitability in children reveals decreased global mean field power and smaller N100 TMS–evoked potential amplitude (23,24). Pediatric TMS-EEG studies have also demonstrated increased complexity and spreading of TMS-evoked EEG waveforms, with decreased consistency throughout childhood (23). These developmental changes are correlated with improvements in manual dexterity, EEG measures of functional connectivity, and cortical maturation indices (23).

TMS has been used to probe intracortical inhibition across development with mixed results; one study found shorter cSPs in children (25), while others have shown no age-related changes (26). Discrepancies may partially arise from differences between motor thresholds of children and adults, leading to different effective stimulation doses (27). Paired-pulse studies have also yielded contradictory results, with early studies showing reduced short-interval intracortical inhibition (SICI) (28) but a more recent study finding no age effect when interstimulus intervals were individually determined, suggesting contamination by concurrent short-interval intracortical facilitation (ICF) in previous studies (29). Limited research exists on rTMS effects in healthy, typically developing children; available studies used a single session of TBS and reported high interindividual variability and no significant age effect for 8- to 17-year-old children (30).

As individuals transition into physiological aging, complex biological changes occur at the molecular, cellular, and system levels. Age-related cortical atrophy can increase the coil-tocortex distance, making RMT a useful measure for examining brain aging (31). Although some studies and meta-analyses have reported increased RMT in older adults, this finding has not been consistently replicated (32). The literature is mixed on whether there is a decrease, no change, or an increase in SICI in older adults (32). A meta-analysis comparing 187 older adults with 169 young adults showed a reduction in SICI in older adults, although the difference was not statistically significant (32). Conversely, ICF has generally been found to be normal in most studies, with a few exceptions (32). A meta-analysis showed a slight, nonsignificant reduction of ICF in older adults (32). Studies on long-interval intracortical inhibition (LICI) have also been inconsistent, with some reporting an increase (33) and others reporting a decrease (34). Consequently, it remains unclear whether GABAergic neurotransmission is generally preserved or impaired in physiological aging (35). Short-latency afferent inhibition (SAI) has been more consistently altered in the aging brain (12,36,37), correlating with age (32), which suggests a progressive decline in cholinergic activity during aging.

TMS-EEG studies investigating age-related changes in motor cortical excitability and global mean field power have shown reduced measures of excitability (38), consistent with MEP findings. Single TMS-evoked potential peaks analysis found decreased local P30 amplitude after M1 stimulation but an opposite trend in ipsilateral prefrontal areas, suggesting true prefrontal hyperexcitability rather than compensatory M1 hypoexcitability (39). The amplitude of the N45 peak was found to be modulated by aging, but with contradictory results (39).

A recent meta-analysis found a general decrease in motor cortex plasticity, but with variability depending on the study and stimulation protocols used (32). Studies on paired associative stimulation–induced long-term potentiation (LTP)–like plasticity of M1 have shown that older adults show less MEP facilitation than younger adults (40). This effect may be more pronounced in women due to hormonal changes that occur during menopause (41). However, studies on M1 plasticity induced by intermittent TBS have shown that M1 excitability increased similarly in both young and older participants (42,43).

In conclusion, it is essential to recognize that the neurophysiological changes that occur from childhood to advanced aging profoundly influence responses to TMS (Table 2). Childhood and adolescence, stages of remarkable brain development and hormonal fluctuations, exhibit distinctive TMS response profiles that require tailored approaches. This complexity extends into adulthood, when neurophysiological changes associated with aging, such as cortical atrophy and shifts in cortical excitability, further modify TMS responses. Additional factors, including sex-specific hormonal variations across different life stages (e.g., puberty, pregnancy, postpartum, and menopause in women), play a significant role in shaping TMS responsiveness. Moreover, lifestyle elements such as diet, physical activity, and sleep patterns significantly affect neurophysiological responses to TMS. All of these facets underscore the need to comprehensively understand the lifespan trajectory of neurophysiological changes to optimize TMS protocols for individual patients while considering their age, sex, and lifestyle factors.

## PEDIATRIC AND NEURODEVELOPMENTAL DISORDERS

Many neurodevelopmental and pediatric neuropsychiatric disorders may be characterized by aberrant cortical and subcortical excitability and/or dysfunctional network connectivity. TMS has been used to probe these mechanisms to identify potential diagnostic and predictive biomarkers and therapeutically through rTMS experimental interventions. However, the literature has several limitations, which consist primarily of small-scale, uncontrolled, or open-label trials and case reports. Most studies have focused on older children and adolescents without intellectual disabilities. Furthermore, protocols trialed in pediatric conditions generally mirror those developed for adult psychiatric populations, thereby neglecting the neurodevelopmental differences reviewed above. Consequently, findings should be interpreted cautiously, and largerscale, blinded, sham-controlled studies are needed prior to broad clinical implementation of these TMS protocols (Table 2).

### Autism Spectrum Disorder

Theories about the pathophysiology of autism spectrum disorder (ASD) suggest an imbalance in excitatory/inhibitory and plasticity/metaplasticity mechanisms (44,45); however, early studies with adolescents and young adults with ASD found no differences compared with typically developing control participants in motor threshold, input-output curves, cSP duration, SICI, or ICF (46,47). It remains unknown whether deficits in these mechanisms exist earlier in development or whether alternative cortical regions or alternative protocols may reveal abnormalities not found in the extant literature. Combining TMS with EEG in children with ASD showed typical developmental profiles for interhemispheric signal propagation (ISP), a measure of interhemispheric connectivity, with no association between ISP and behavioral symptoms (48). TBS studies have found greater, longer-lasting effects on motor cortical excitability in children with ASD, with aberrant responses becoming more pronounced with age (30,49–51). Although TBS studies suggest a hyperplastic response, one study found reduced LTP-like plasticity in ASD using paired associative stimulation (52), which was not associated with SICI measures of GABAergic transmission (52).

Various rTMS protocols have been trialed in ASD. Low-frequency rTMS applied over the dorsolateral prefrontal cortex (DLPFC) (53–55) and intermittent TBS over the posterior superior temporal cortex (56) improved social relating and reduced repetitive behaviors. Higher IQ, better baseline social cognitive performance, less severe social-communicative impairments, less severe baseline ASD symptoms, and less attention-deficit/hyperactivity disorder (ADHD) severity predicted better response to posterior superior temporal cortex stimulation for social relating (56). Low-frequency stimulation over the DLPFC also improved executive functioning (57), irritability (54), hyperactivity (54), and cooccurring depression (58). In addition, 8-Hz stimulation over premotor cortex improved sensorimotor integration (59), while 5-Hz stimulation over the right inferior frontal gyrus combined with action observation and execution improved adaptive behaviors (60).

### ADHD and Tourette Syndrome

Children with ADHD and Tourette syndrome (TS) showed deficits in response inhibition and motor control (61). Early studies found no difference between children with ADHD and typically developing children regarding RMT, MEP amplitudes, cSP, or ICF (62,63). However, a recent study revealed enhanced ICF with an interstimulus interval of 15 ms and a longer cSP duration (64). In children with TS, early studies found a shortened cSP with no difference in RMT between children with TS and typically developing control participants, and neither measure was correlated with clinical symptom severity (65,66). A more recent study suggested that children with TS had higher RMT with a shallower input-output curve with increasing intensities, normalizing with age over adolescence (67).

Consistent with a deficit in inhibitory control and dysfunctional network connectivity, children with ADHD showed longer ISP and ipsilateral cortical silent period latencies and shorter ISP durations (63,68). However, the duration and latency of the ISP was not correlated with either age or symptom severity (63). Children with ADHD also showed reduced intracortical inhibition (62,69), and children with ADHD and TS showed less task-related upmodulation immediately preceding a motor response and during a response inhibition task (69–71). The degree of reduction in task-related upmodulation was correlated with motor tic severity in TS, and the degree of SICI, ipsilateral cortical silent period latency, and task-related upmodulation were negatively correlated with ADHD symptom severity and ratings on a motor impairment scale (68,69). In children with TS, no evidence of aberrant ISP was found (70), but SICI seemed reduced in TS and was negatively correlated with tic severity (66).

TMS-EEG studies in children with ADHD have yielded mixed results. One study found a smaller TMS-evoked N100 potential with a shorter latency in children with ADHD (72), but this finding was not replicated in a later study (73). When N100 was evaluated for a concurrent task, children with ADHD showed reduced modulation by response preparation or movement execution, with a decrease in amplitude with increased age (72,73). A recent trial found that 1-Hz rTMS led to an unexpected reduction in the TMS-evoked N100 amplitude (74).

The rTMS therapeutic literature on ADHD and TS is similarly mixed in terms of protocols and efficacy. While 1-Hz rTMS over the left DLPFC improved inattentiveness and hyperactivity/impulsivity in children with ADHD (75), 10-Hz stimulation to the right DLPFC did not differentially improve ADHD symptoms compared with sham stimulation (76). For TS, all studies applied either 1-Hz rTMS or continuous TBS intended to inhibit the supplementary motor area. Open-label trials with small samples have reported positive effects with improvements in TS symptom severity (77,78), with one study correlating with an increase in RMT (77). A single randomized, sham-controlled trial of continuous TBS found no difference in improvement of TS symptom severity between active and sham stimulation, with both showing significant improvements from baseline to posttreatment (79).

### Adolescent Depression

In adolescent depression, single- and paired-pulse paradigms have revealed enhanced ICF, but no difference in RMT, cSP, or paired-pulse intracortical inhibition measures (80). However, a more recent study found reduced SICI before antidepressant treatment, and an increase in SICI was associated with improvement in symptom severity at follow-up (81). A significant age effect on both RMT and LICI was reported (82). In addition, a shorter duration of cSP (reduced inhibition) was related to higher depression scores (83), and less LICI (reduced inhibition) predicted nonresponse to fluoxetine treatment (84).

Contrary to motor cortex findings, a recent study found that TMS-evoked EEG markers of cortical inhibition and reactivity (N100 and P200) increased in depressed adolescents when TMS was applied over the DLPFC, but not over the motor cortex or inferior parietal lobule sites. Anhedonia was negatively correlated with P200 amplitude (85). The same group investigated TMS-evoked EEG potentials before and after a course of rTMS and found that baseline N45 (a marker of GABA_A_ neurotransmission) over frontal sites predicted clinical treatment response, and modulation of N45 potentials over inferior parietal lobule was correlated with the degree of functional connectivity between the DLPFC and inferior parietal lobule (86).

Early case reports and small open-label trials of rTMS in adolescent depression have shown therapeutic promise, with relatively large effect sizes and average reduction in depressive symptoms ranging from 23% to 71% (87,88). Most studies applied 10-Hz unilateral left DLPFC rTMS, largely mimicking adult studies. Two exceptions include one study that compared 1-Hz, unilateral right DLPFC stimulation with bilateral stimulation (89) and another that applied bilateral TBS stimulation (90). However, the only large-scale, randomized, sham-controlled trial that applied the standard adult depression protocol reported an effect size near 0. Both active and sham rTMS groups experienced a reduction in depressive symptoms, with response and remission rates of 41.7% and 29.2% for the active group and 36.4% and 29% for the sham group, respectively (91).

### Adolescent Anxiety Disorders

Although rTMS has been extensively studied in adult anxiety disorders including obsessive-compulsive disorder, posttraumatic stress disorder, and generalized anxiety disorder, there is a paucity of studies in pediatric populations. In fact, the only study that has examined the effects of TMS in patients with a primary anxiety disorder is a single-session rTMS study in adolescents with obsessive-compulsive disorder. This study examined the effects of a single session of 1-Hz rTMS (1800 pulses applied over the right DLPFC at 110% of RMT) and found no change in functional magnetic resonance imaging blood oxygen level–dependent response from pre- to postsession in the corticostriatal-thalamic circuit (92). Thus, there is an unmet need to examine the effects of various TMS protocols in youth.

## AGING AND NEURODEGENERATIVE DISORDERS

Neurodegenerative disorders, including Alzheimer’s disease (AD), dementia with Lewy bodies (DLB), Parkinson’s disease, and frontotemporal dementia (FTD), are age-related conditions characterized by the progressive degeneration of nerve cells in the brain, resulting in decline of cognitive and motor functions. Importantly, late-onset/geriatric depression often coexists with these disorders and may represent a prodromal symptom or act as risk factor, suggesting a potential reverse causation pathway in the development of these neurodegenerative conditions. TMS has been used to assess motor cortex excitability, which is modulated by several neurotransmitter circuits, many of which are affected in these conditions (Table 2).

### Late-Onset/Geriatric Depression

In individuals with late-onset/geriatric depression, TMS has revealed significant neurophysiological changes. Alterations in the RMT and cSP have been reported, indicating potential disruptions in inhibitory GABAergic neurotransmission (93). Furthermore, a decrease in SICI and an increase in ICF, reflecting glutamatergic hyperactivity, have been documented (93).

In the context of therapeutic applications, high-frequency rTMS over the left DLPFC has been shown to mitigate depressive symptoms in late-onset depression (94). These effects are believed to stem from the modulation of neuroplasticity and the enhancement of neural connectivity within mood-regulating networks (94). Recent studies have also indicated potential predictive markers for treatment response, such as baseline motor cortex excitability and the presence of specific genetic polymorphisms (95).

### Alzheimer’s Disease

In AD, TMS has proven useful in evaluating cortical network functionality (96). Research indicates increased motor cortex excitability in AD as evidenced by reduced RMT and active motor threshold (96). This change reflects the complex interplay between GABAergic, glutamatergic, and cholinergic neurotransmission in M1, leading to an imbalance between excitatory and inhibitory activities (97). Some studies found no change in SICI, while others reported a reduction (96,98–100). A meta-analysis found that SICI reduction occurred in patients with AD with longer symptom duration (101). Most studies found no change in cSP duration (102,103), but a few reported a reduction in LICI (100,104). Reduced ICF was observed in AD (96,99,100,104), whereas SAI was significantly decreased (96,104–107). Interhemispheric connectivity, measured by ISP, was impaired in patients with AD, resulting in prolonged latencies (108), and posterior parietal cortex–M1 connectivity was also impaired (109). Patients with AD displayed weakened LTP-like cortical plasticity compared with healthy participants (96,110).

TMS-EEG has been used to explore brain circuits in AD, revealing alterations in frontoparietal and sensorimotor pathways. Stimulation of the frontal cortex in patients with AD exposed local signal propagation and cortical excitability dysfunctions (111) and increased effective connectivity between the prefrontal and contralateral parietal regions, as measured by the P30 TMS-evoked potential component (112). In the early stages of AD, TMS-EEG over M1 shows P30 alterations, indicating abnormal activity and connectivity in the sensorimotor system (113). Patients with AD also exhibit a significant reduction in frontal gamma connectivity (106,114).

Recent studies have demonstrated effectiveness of rTMS in enhancing cognitive performance, memory, and executive functions in patients with AD (115). High-frequency rTMS over the left DLPFC has shown potential in improving cognitive function, with some studies reporting effects up to 6 months after treatment (116). In addition, precuneus rTMS modulates AD-related biomarkers such as cortical excitability and synaptic plasticity (117). Although the precise mechanisms underlying TMS-induced improvements in AD remain unclear, these findings suggest that rTMS may be a valuable addition to current pharmacological interventions.

### Dementia With Lewy Bodies

DLB is a neurodegenerative disorder that is characterized by cognitive impairment, visual hallucinations, and motor dysfunction. Reports on SICI in DLB have produced inconsistent results. While some studies did not find any differences in SICI between patients with DLB and control participants (96), 3 other studies found reduced SICI and ICF in patients with DLB compared with patients with AD and healthy control participants (104,118–120), which was correlated with motor impairment. SAI has been studied more extensively in DLB due to its profound cholinergic dysfunction. Generally, patients with DLB tend to show reduced SAI compared with healthy control participants (96,99,121,122), and reduced SAI is correlated with visual hallucination severity (122) and cognitive decline (123). Only one study has evaluated the effects of DLPFC rTMS in 6 patients with DLB with drug-resistant depression, showing significantly improved depressive symptoms after treatment (124).

### Parkinson’s Disease

TMS has provided unique insights into the neurophysiological alterations associated with Parkinson’s disease, particularly those related to cognitive and neuropsychiatric dimensions of the disorder. Patients with Parkinson’s disease typically present with reduced cortical plasticity, demonstrated by impaired LTP and long-term depression–like plasticity induction through paired associative stimulation and TBS protocols (125), with an imbalance of inhibitory (SICI) and excitatory (ICF) circuits (125). Alterations in SAI have also been observed, suggesting that cholinergic degeneration may be an important contributor to the clinical features of the disease, particularly nonmotor symptoms and cognitive impairment (125).

Concerning therapeutic applications, rTMS has shown promise in ameliorating cognitive deficits and neuropsychiatric symptoms associated with Parkinson’s disease. High-frequency rTMS over the DLPFC has been shown to enhance executive functions and working memory, potentially linked to increased synaptic plasticity and dopaminergic function in the prefrontal-basal ganglia-cortical circuits (126). Furthermore, rTMS delivered to the left DLPFC may improve depressive symptoms (127).

### Frontotemporal Dementia

FTD is a common neurodegenerative disorder that is characterized by behavioral abnormalities, language impairment, and executive function deficits. Patients with FTD exhibit motor circuit abnormalities, including decreased M1 excitability, absent MEPs, increased MEP latencies, and central motor conduction time (96). Studies have also shown significant reductions in SICI, LICI, and ICF, reflecting GABAergic and glutamatergic abnormalities that are typical of FTD pathology (118,128–134). However, SAI levels are typically normal in FTD (96,121,135–139). SICI and ICF are significantly associated with behavioral symptoms in patients with FTD, with positive symptoms correlating with reduced SICI and LICI and negative symptoms linked to decreased ICF (118). TMS measures can predict disease severity, with SICI being the best predictor of FTD progression (135). LTP-like plasticity is reduced in both presymptomatic and symptomatic FTD (138). Studies investigating TMS combined with cognitive training have shown promising results, suggesting a synergistic effect enhancing the benefits of each intervention (140).

## CONCLUSIONS/FUTURE DIRECTIONS

TMS has emerged as a powerful tool for understanding the neurophysiological underpinnings of neuropsychiatric, neurodevelopmental, and neurodegenerative disorders across the lifespan. By elucidating the changes in physiology that occur in healthy development/aging and disease, TMS can inform diagnostic and therapeutic approaches. Clinical applications range from early detection and diagnosis to targeted interventions and personalized treatment. By harnessing the power of TMS to modulate cortical excitability and neuroplasticity, we can develop innovative strategies for combating the debilitating effects of neurodevelopmental and neurodegenerative disorders. Integration with other therapeutic modalities may lead to more comprehensive and effective treatment plans that address the multifaceted nature of these conditions.

Unanswered questions remain, including questions about the ability of TMS to predict the onset of neurodevelopmental or neurodegenerative disorders. Longitudinal studies are needed to establish whether early TMS markers can reliably identify individuals at risk, thereby enabling preventive measures before overt symptoms manifest. In addition, the long-term effects of TMS on neural circuits and their relationship to clinical outcomes warrant further investigation. Longitudinal TMS studies tracking neurotransmitter circuits and cortical plasticity development will enable a better understanding of disease progression with and without therapeutic interventions.

The heterogeneity of clinical presentations poses challenges for identifying consistent TMS-based biomarkers. Integrating TMS with other neuroimaging and neurophysiological techniques may provide a more holistic view of the neural mechanisms underpinning these disorders. Advanced data analytic techniques, such as machine learning algorithms, may aid in identifying robust TMS-based biomarkers and developing personalized treatment strategies. TMS may play a critical role in tailoring interventions to an individual’s unique risk profile by integrating genetic data, neuroimaging findings, and other clinical information. Personalizing treatments to the individual’s current neurophysiological brain state may reduce within-group variability and increase effect sizes.

Understanding the underlying neural mechanisms of TMS is vital for maximizing its diagnostic and therapeutic potential. Although the modulation of cortical excitability and neuroplasticity is well documented, the precise mechanisms by which TMS affects the developing and aging brain remain unclear. Future research should focus on uncovering these mechanisms, thereby facilitating more targeted and individualized TMS interventions. Interdisciplinary collaborations among neuroscientists, engineers, clinicians, and stakeholders will be crucial for refining TMS technology and methodology to enhance its effectiveness and minimize potential side effects.

As TMS techniques continue to advance and their application expands, it is imperative to rigorously evaluate their safety and efficacy in these special populations. Future research should prioritize comprehensive long-term follow-up studies involving larger cohorts to assess potential cognitive and neural impacts of TMS in pediatric and geriatric patients. These approaches will ensure that TMS research remains at the forefront of neurological science, ultimately leading to improved outcomes for individuals affected by neurodevelopmental and neurodegenerative disorders.

## Supplementary Material

Supplementary Material

## Figures and Tables

**Table 1.** Summary of Main TMS Protocols: An Overview of Mechanisms, Neuroimaging or Neurophysiology Outcomes, Associated Receptor and Neurotransmitter Systems, and Relevant Populations/Disorders

TMS Protocol	Mechanism Probed	Neuroimaging or Neurophysiology Outcome^a^	Associated Receptor and Neurotransmitter Systems	Relevant Populations/Disorders Based on Developmental Physiology and Pathophysiology
MEPs and Motor Threshold	Neuronal membrane excitability	EMG recorded with the target muscle at rest (resting motor threshold) or during a slight tonic contraction at approximately 20% of the maximal muscle strength (active motor threshold)	Non-NMDA glutamatergic transmission	Developmental and aging-related changes in myelination and glutamatergic transmission
Central Motor Conduction Time	Integrity of the corticospinal tract	EMG estimated by subtracting the conduction time from the spinal roots/nerves to the muscle from the latency of MEPs evoked by TMS delivered during voluntary contraction of the target muscle	Non-NMDA glutamatergic transmission	Motor neuron disease, myelopathy, and FTD
Contralateral Cortical Silent Period	Intracortical inhibition	EMG recorded 100–300 ms after TMS pulse during contralateral voluntary muscle contraction or EEG	GABA_B_, dopamine transmission	Typical development
Ipsilateral Cortical Silent Period	Interhemispheric signal propagation	EMG recorded during ipsilateral voluntary muscle contraction or EEG	GABA_B_	ADHD, AD
Paired-Pulse TMS With 1.3- to 1.5-ms ISI	Short-interval intracortical facilitation	EMG recorded by applying a suprathreshold TMS conditioning stimulus and a subthreshold or threshold TMS test stimulus	Non-NMDA glutamatergic transmission	Typical development and PD
Paired-Pulse TMS With 1- to 6-ms ISI	Short-interval intracortical inhibition	EMG recorded by applying a subthreshold TMS conditioning stimulus and a suprathreshold TMS test stimulus	GABA_A_ transmission	Typical development, healthy aging, ADHD, Tourette syndrome, adolescent depression, late-onset depression, AD, DLB, PD, FTD
Paired-Pulse TMS With 6- to 30-ms ISI	Intracortical facilitation		NMDA glutamatergic transmission	Healthy aging, ADHD, adolescent depression, late-onset depression, AD, DLB, PD, FTD
Paired-Pulse TMS With 50- to 200-ms ISI	Long-interval intracortical inhibition	EMG recorded by applying a suprathreshold TMS conditioning stimulus and a suprathreshold TMS test stimulus	GABA_B_ transmission	AD, FTD
Single-Pulse TMS Combined With Peripheral Electrical Stimulation With a 20- to 28-ms ISI	Short-latency afferent inhibition	EMG recorded by applying a conditioning electrical stimulation at the median nerve and a suprathreshold TMS test stimulus	Acetylcholine and GABA transmission	Healthy aging, AD, DLB, PD
Repeated Pairings of Single-Pulse TMS Combined With Peripheral Electrical Stimulation With a 10-ms ISI	LTD-like associative plasticity	EMG recorded by applying repeated electrical stimulations at the median nerve and suprathreshold TMS test stimuli after 10 ms	NMDA glutamatergic transmission	DLB
Repeated Pairings of Single-Pulse TMS Combined With Peripheral Electrical Stimulation With a 25-ms ISI	LTP-like associative plasticity	EMG recorded by applying repeated electrical stimulations at the median nerve and suprathreshold TMS stimuli after 25 ms	NMDA glutamatergic transmission	Healthy aging, ASD, AD, DLB, FTD
Bursts of 3 Pulses of Gamma Frequency (30–50 Hz) Stimulation With a 200-ms Interburst Interval Repeated With (Intermittent) or Without (Continuous) Intertrain Intervals (Theta Burst Stimulation)	Non-Hebbian LTP-like (intermittent theta burst) and LTD-like (continuous theta burst) plasticity	EMG or EEG	NMDA glutamatergic and GABAergic transmission	ASD

AD, Alzheimer’s disease; ADHD, attention-deficit/hyperactivity disorder; ASD, autism spectrum disorder; DLB, dementia with Lewy bodies; EEG, electroencephalography; EMG, electromyography; FTD, frontotemporal dementia; GABA, gamma-aminobutyric acid; ISI, interstimulus interval; LTD, long-term depression; LTP, long-term potentiation; MEP, motor evoked potential; PD, Parkinson’s disease; TMS, transcranial magnetic stimulation.

a EMG is recorded at the peripheral nerve (typically on the hand).

**Table 2.** Summary of TMS Findings and Therapeutic Applications in Normal Development, Healthy Aging, Pediatric and Neurodevelopmental Disorders, and Adult-Onset Neurodegenerative Conditions

Condition and Relevant TMS Findings	Therapeutic Applications

Typical Development	
cSP ↓ ⟷ (25,26)	
LTP-iTBS ⟷ (30)	
MEP ↓ (23,24)	
RMT ↑ (21,22)	
SICI ⟷ ↓ (28,29)	
N100 ↓ (23,24)	

Healthy Aging	
ICF ⟷ ↓ (32)	
LICI ⟷ (33,34)	
LTP-iTBS ⟷ (32,42,43)	
LTP-PAS ↓ (32,40,41)	
RMT ⟷ ↑ (31,32)	
SAI ↓ (12,32,36,37)	
SICI ⟷ ↓ (32)	
P30 ↓ (39)	
N45 ⟷ (39)	

Autism Spectrum Disorder	Low-frequency rTMS over DLPFC (53–55,57,58), over rIFG (60), and iTBS over pSTS (56) improved behavior; 8-Hz stimulation over premotor cortex improved sensorimotor integration (59)
cSP ⟷ (46,47)	
SICI ⟷ (46,47)	
ICF ⟷ (46,47)	
LTP-TBS ↑ (30,49–51)	
LTP-PAS ↓ (52)	

Attention-Deficit/Hyperactivity Disorder	1-Hz rTMS over DLPFC improves attentiveness and hyperactivity/ impulsivity (75)
cSP ⟷ ↑ (62,63)	
ICF ⟷ ↑ (62–64)	
ISP ↑ (63,68)	
MEP ⟷ (62,63)	
RMT ⟷ (62,63)	
SICI ↓ (62,69)	
N100 ↓ ⟷ (72,73)	

Tourette Syndrome	1-Hz rTMS or cTBS over SMA improves symptom severity (77,78)
cSP ↓ (65,66)	
RMT ⟷ ↑ (65–67)	
SICI ↓ (66)	

Adolescent Depression	rTMS over DLPFC reduces depressive symptoms (87,88)
cSP ⟷ ↓ (80,83)	
ICF ↑ (80)	
RMT ⟷ (80,82)	
SICI ⟷ ↓ (80,81)	
N100 ↑ (85)	
P200 ↑ (85)	

Late-Onset Depression	rTMS over DLPFC reduces depressive symptoms (94)
cSP ↓ (93)	
ICF ↑ (93)	
RMT ↓ (93)	
SICI ↓ (93)	

Alzheimer’s Disease	rTMS over DLPFC (116) and precuneus (117) enhances cognitive performance, memory, and executive functions
AMT ↓ (96)	
cSP duration ⟷ (102,103)	
RMT ↓ (96)	
ICF ⟷ ↓ (96,99,100,104)	
ISP ↓ (108)	
LICI ⟷ ↓ (96,100,104)	
LTP ↓ (96,110)	
LTD ⟷ (96)	
SAI ↓ (96,104–107)	
SICI ⟷ ↓ (96,98–101)	
P30 ↑ (112)	

Dementia With Lewy Bodies	rTMS improves depressive symptoms (124)
ICF ↓ (96,104,118–120)	
SAI ↓ (96,99,121–123)	
SICI ↓ (96,104,118–120)	

Parkinson’s Disease	rTMS over DLPFC enhances cognitive functions (126) and improves depressive symptoms (127)
cSP duration ⟷ ↓ (125)	
ICF ⟷ ↓ (125)	
LTP ↓ (125)	
LTD ↓ (125)	
RMT ⟷ ↓ (125)	
SAI ⟷ ↓ (125)	
SICI ↓ (125)	
SICF ↑ (125)	

Frontotemporal Dementia	rTMS improves language in primary progressive aphasias (140)
CMCT ↑ (96)	
ICF ↓ (118,128–134)	
RMT ⟷ (96)	
LICI ↓ (118,128–134)	
LTP ↓ (138)	
SAI ⟷ (96,121,135–139)	
SICI ↓ (118,128–135)	

↑, increased; ⟷, no change or normal; ↓, reduced; AMT, active motor threshold; CMCT, central motor conduction time; cSP, cortical silent period; cTBS, continuous TBS; DLPFC, dorsolateral prefrontal cortex; ICF, intracortical facilitation; ISP, interhemispheric signal propagation; iTBS, intermittent TBS; LICI, long-interval intracortical inhibition; LTD, long-term depression; LTP, longterm potentiation; MEP, motor evoked potential; PAS, paired associative stimulation; pSTS, posterior superior temporal cortex; rIFG, right inferior frontal gyrus; RMT, resting motor threshold; rTMS, repetitive TMS; SAI, short-latency afferent inhibition; SICF, short-interval intracortical facilitation; SICI, short-interval intracortical inhibition; SMA, supplementary motor area; TBS, theta burst stimulation; TMS, transcranial magnetic stimulation.
