# Supplementary material for: Transcranial Magnetic Stimulation Across the Lifespan: Impact of Developmental and Degenerative Processes
Source: Biol Psychiatry. Author manuscript; Available in PMC 2024 Mar 15. (PMC10823041; doi:10.1016/j.biopsych.2023.07.012)
Supplement: Supplementary Material [file NIHMS1941759-supplement-Supplementary_Material.docx]

**SUPPLEMENTARY INFORMATION**

**Transcranial Magnetic Stimulation Across the Lifespan: Impact of Developmental and Degenerative Processes**

Oberman and Benussi

# **TMS Protocols**

## *Paired-Pulse TMS*

Paired-pulse TMS has been widely used to investigate excitability and intracortical inhibition throughout the lifespan. Short interval intracortical inhibition (SICI) is elicited when a subthreshold conditioning stimulus is followed by a suprathreshold test stimulus at an interstimulus interval (ISI) of 1-6 ms, which reflects intracortical post-synaptic inhibition mediated by GABA_A_ receptors [1, 2]. Intracortical facilitation (ICF) is elicited similarly as SICI but at longer ISIs of 6-30 ms, reflecting excitatory NMDA-glutamatergic circuits [3, 4]. Short interval intracortical facilitation (SICF) can be induced through a paired-pulse protocol where the first stimulus is suprathreshold and the second stimulus is delivered at approximately the resting motor threshold (RMT) with an ISI of 1.3–1.5 ms, which reflects the activity of non-NMDA glutamatergic neurotransmission [5]. Long interval intracortical inhibition (LICI) is tested by applying two suprathreshold stimuli at long ISIs of 50-200 ms and is mediated by GABA_B_ receptors [6]. Short latency afferent inhibition (SAI) is based on a sensorimotor cortical integration process, in which the response to a magnetic test pulse on the motor cortex is inhibited by a conditioning afferent stimulus of a sensory nerve, typically the median or ulnar nerve at the wrist, and depends on cholinergic and GABAergic circuits [7].

## *Paired-Associative Stimulation (PAS)*

In addition to probing excitatory or inhibitory tone, TMS can also be used to study corticospinal and cortico-cortical plasticity. One such protocol involves applying paired-associative stimulation protocols (PAS), which includes electrical stimulation of the peripheral median nerve repeatedly paired with a TMS stimulus over the contralateral motor cortex [8]. PAS with an ISI of 25 ms can lead to facilitation of motor evoked potentials (MEPs), mainly dependent on NMDA receptors, whereas an ISI of 10 ms results in inhibition [9]. These effects respectively represent a form of associative long-term potentiation-like (LTP-like) and long-term depression-like (LTD-like) plasticity [10]. Outside of the motor cortex, similar protocols are also being developed to probe cortico-cortical plasticity [11].

*Repetitive Transcranial Magnetic Stimulation (rTMS)*

TMS can also be applied in trains of regularly repeating TMS pulses (i.e., repetitive TMS or rTMS). rTMS can be applied at various stimulation frequencies (e.g., 1-10 Hz) and patterns (e.g., Theta Burst Stimulation (TBS) [12]). Compared to paired-pulse or single-pulse stimulation protocols, rTMS pulses temporally summate to produce longer lasting changes in neural activity [13]. The effects of a single session of rTMS can last minutes to hours, while effects from repeated sessions can last weeks to months. Stimulation frequencies of ≤1 Hz generally induce local cortical inhibition while frequencies ≥5 Hz generally induce local cortical excitation [13]. Patterned forms of rTMS, including intermittent theta burst stimulation (iTBS) and continuous TBS (cTBS), are thought to lead to long-lasting facilitation and suppression of cortical excitability respectively via non-Hebbian LTP-like and LTD-like mechanisms.

# **Safety of TMS Across the Lifespan**

The safety of rTMS in clinical practice and research has been evaluated through multiple meta-analyses (e.g., [14, 15]). Safety guidelines have been disseminated by the International Federation of Clinical Neurophysiology [16]. Widespread application of several rTMS protocols, across diverse populations and devices, show a low incidence of serious adverse events [17]. TMS protocols have been reported to have a very low risk of inducing seizures, with a seizure incident rate of <0.01% [16]. Though previous TMS Safety guidelines [18] advised caution in the application of TMS in persons taking medications known to lower seizure threshold (many of which are commonly prescribed in pediatric and geriatric Psychiatric disorders, currently available data showing low seizure rate no longer support this recommendation.

Most of the safety literature in TMS is based on young and middle-aged adults. Compared to the young adult literature, data on the safety of rTMS in pediatric and geriatric populations are relatively lacking. The neurodevelopmental and aging processes ongoing in children, adolescents, and older adults, compounded by the pathophysiological processes affecting those with neuropsychiatric, neurodevelopmental, and neurodegenerative disorders, require careful consideration[19-21]. Existing data suggest a similar safety profile in children, adolescents, and geriatric populations as compared to young adults with the prevalence of mild adverse event rates ranging from 3.4% to 10.11% and serious adverse event (seizures and syncope) occurring in less than 1% of patients (even in disorders with higher seizure risk, e.g., epilepsy, stroke, and neurodevelopmental disorders). [22-25]. The most recent International Federation of Clinical Neurophysiology TMS safety guidelines indicate that the extant pediatric literature “provides reassurance regarding the safety of these techniques” in pediatric populations [16]. However, this “reassurance” is based on far less data than in the adult literature.

With respect to possible unexpected long-term effects, particularly in pediatric populations, the current state of knowledge is largely insufficient. Children's brains undergo significant structural and functional changes, and the long-term impact of TMS on this dynamic neurodevelopmental process is unclear [26]. Preliminary studies suggest no significant negative effects on cognitive function or behavior, but these are mostly based on short-term follow-ups and small sample sizes [22].

**References**

1. Kujirai, T., et al., *Corticocortical inhibition in human motor cortex.* J Physiol, 1993. 471: p. 501-19.

2. Di Lazzaro, V., et al., *Segregating two inhibitory circuits in human motor cortex at the level of GABAA receptor subtypes: a TMS study.* Clin Neurophysiol, 2007. 118(10): p. 2207-14.

3. Ziemann, U., J.C. Rothwell, and M.C. Ridding, *Interaction between intracortical inhibition and facilitation in human motor cortex.* J Physiol, 1996. 496 ( Pt 3)(Pt 3): p. 873-81.

4. Di Lazzaro, V., et al., *Origin of facilitation of motor-evoked potentials after paired magnetic stimulation: direct recording of epidural activity in conscious humans.* J Neurophysiol, 2006. 96(4): p. 1765-71.

5. Ziemann, U., et al., *Demonstration of facilitatory I wave interaction in the human motor cortex by paired transcranial magnetic stimulation.* J Physiol, 1998. 511 ( Pt 1)(Pt 1): p. 181-90.

6. Valls-Sole, J., et al., *Human motor evoked responses to paired transcranial magnetic stimuli.* Electroencephalogr Clin Neurophysiol, 1992. 85(6): p. 355-64.

7. Tokimura, H., et al., *Short latency inhibition of human hand motor cortex by somatosensory input from the hand.* J Physiol, 2000. 523 Pt 2(Pt 2): p. 503-13.

8. Stefan, K., et al., *Mechanisms of enhancement of human motor cortex excitability induced by interventional paired associative stimulation.* The Journal of physiology, 2004. 543(2): p. 699-708.

9. Wolters, A., *A Temporally Asymmetric Hebbian Rule Governing Plasticity in the Human Motor Cortex.* Journal of Neurophysiology, 2003. 89(5): p. 2339-2345.

10. Stefan, K., et al., *Induction of plasticity in the human motor cortex by paired associative stimulation.* Brain, 2000. 123 Pt 3: p. 572-84.

11. Thut, G., et al., *Guiding transcranial brain stimulation by EEG/MEG to interact with ongoing brain activity and associated functions: A position paper.* Clin Neurophysiol, 2017. 128(5): p. 843-857.

12. Huang, Y.Z., et al., *Theta burst stimulation of the human motor cortex.* Neuron, 2005. 45(2): p. 201-6.

13. Hallett, M., *Transcranial magnetic stimulation and the human brain.* Nature, 2000. 406(6792): p. 147-50.

14. Janicak, P.G., et al., *Transcranial magnetic stimulation in the treatment of major depressive disorder: a comprehensive summary of safety experience from acute exposure, extended exposure, and during reintroduction treatment.* J Clin Psychiatry, 2008. 69(2): p. 222-32.

15. Machii, K., et al., *Safety of rTMS to non-motor cortical areas in healthy participants and patients.* Clin Neurophysiol, 2006. 117(2): p. 455-71.

16. Rossi, S., et al., *Safety and recommendations for TMS use in healthy subjects and patient populations, with updates on training, ethical and regulatory issues: Expert Guidelines.* Clin Neurophysiol, 2021. 132(1): p. 269-306.

17. Lerner, A.J., E.M. Wassermann, and D.I. Tamir, *Seizures from transcranial magnetic stimulation 2012-2016: Results of a survey of active laboratories and clinics.* Clin Neurophysiol, 2019. 130(8): p. 1409-1416.

18. Rossi, S., et al., *Safety, ethical considerations, and application guidelines for the use of transcranial magnetic stimulation in clinical practice and research.* Clin Neurophysiol, 2009. 120(12): p. 2008-2039.

19. Oberman, L.M. and P. Enticott, eds. *Neurotechnology and Brain Stimulation in Pediatric Psychiatric and Neurodevelopmental Disorders*. 2019, Elsevier Academic Press: Cambridge.

20. Kirton, A. and D.L. Gilbert, eds. *Pediatric Brain Stimulation: Mapping and Modulating the Developing Brain*. 2016, Elsevier Academic Press: San Diego.

21. Iriarte, I.G. and M.S. George, *Transcranial Magnetic Stimulation (TMS) in the Elderly.* Curr Psychiatry Rep, 2018. 20(1): p. 6.

22. Allen, C.H., B.M. Kluger, and I. Buard, *Safety of Transcranial Magnetic Stimulation in Children: A Systematic Review of the Literature.* Pediatr Neurol, 2017. 68: p. 3-17.

23. Zewdie, E., et al., *Safety and tolerability of transcranial magnetic and direct current stimulation in children: Prospective single center evidence from 3.5 million stimulations.* Brain Stimul, 2020. 13(3): p. 565-575.

24. Cappon, D., et al., *Transcranial magnetic stimulation (TMS) for geriatric depression.* Ageing Res Rev, 2022. 74: p. 101531.

25. Overvliet, G.M., et al., *Adverse events of repetitive transcranial magnetic stimulation in older adults with depression, a systematic review of the literature.* Int J Geriatr Psychiatry, 2021. 36(3): p. 383-392.

26. Krishnan, C., et al., *Safety of noninvasive brain stimulation in children and adolescents.* Brain Stimul, 2015. 8(1): p. 76-87.
